# The association of urinary cadmium with sex steroid hormone concentrations in a general population sample of US adult men

**DOI:** 10.1186/1471-2458-8-72

**Published:** 2008-02-23

**Authors:** Andy Menke, Eliseo Guallar, Meredith S Shiels, Sabine Rohrmann, Shehzad Basaria, Nader Rifai, William G Nelson, Elizabeth A Platz

**Affiliations:** 1 Department of Epidemiology, Johns Hopkins Bloomberg School of Public Health, Baltimore, USA; 2 Department of Cardiovascular Epidemiology and Population Genetics, Centro Nacional de Investigaciones Cardiovasculares (CNIC), Madrid, Spain; 3 Division of Cancer Epidemiology, German Cancer Research Center, Heidelberg, Germany; 4 Department of Medicine, Johns Hopkins School of Medicine, Baltimore, USA; 5 Department of Laboratory Medicine, Children's Hospital Boston, Boston, USA; 6 Department of Oncology, Johns Hopkins School of Medicine, Baltimore, USA; 7 Department of Pathology, Johns Hopkins School of Medicine, Baltimore, USA; 8 Department of Pharmacology and Molecular Sciences, Johns Hopkins School of Medicine, Baltimore, USA; 9 Department of Urology, Johns Hopkins School of Medicine, Baltimore, USA; 10 The Sidney Kimmel Comprehensive Cancer Center, Johns Hopkins Medical Institutions, Baltimore, USA; 11 The James Buchanan Brady Urological Institute, Johns Hopkins Medical Institutions, Baltimore, USA

## Abstract

**Background:**

Studies investigating the association of cadmium and sex steroid hormones in men have been inconsistent, but previous studies were relatively small.

**Methods:**

In a nationally representative sample of 1,262 men participating in the morning examination session of phase I (1998–1991) of the third National Health and Nutrition Examination Survey, creatinine corrected urinary cadmium and serum concentrations of sex steroid hormones were measured following a standardized protocol.

**Results:**

After adjustment for age and race-ethnicity, higher cadmium levels were associated with higher levels of total testosterone, total estradiol, sex hormone-binding globulin, estimated free testosterone, and estimated free estradiol (each p-trend < 0.05). After additionally adjusting for smoking status and serum cotinine, none of the hormones maintained an association with urinary cadmium (each p-trend > 0.05).

**Conclusion:**

Urinary cadmium levels were not associated with sex steroid hormone concentrations in a large nationally representative sample of US men.

## Background

Cadmium is a widespread toxic and carcinogenic metal with numerous adverse health effects in humans [1]. In the general population, environmental exposure to cadmium occurs primarily through smoking, the consumption of contaminated food and water, and inhalation of contaminated air [2,3].

Cadmium shows androgen and estrogen-like activities in vitro and in vivo, and it disrupts the male endocrine system in animal models. There is conflicting evidence regarding whether cadmium increases or decreases testosterone production in experimental models [4-6], with decreases more commonly seen after a single, large dose injection of cadmium and increases more commonly seen after chronic oral cadmium exposure [7]. Studies investigating the association of cadmium and sex steroid hormones in men have been inconsistent, finding either no association [8-10], or a positive association between cadmium and testosterone levels [11-13]. Inconsistencies in these studies may be due to small sample sizes, to differences in study design, or to inadequate control of confounders such as tobacco use, a major source of cadmium [14] that is also associated with higher testosterone levels in men [15,16].

The purpose of the current analysis was to evaluate the association of urinary cadmium levels, a biomarker of long term and ongoing cadmium exposure, with serum concentrations of sex steroid hormones (total testosterone, total estradiol, androstanediol glucuronide [AAG], estimated free testosterone, and estimated free estradiol) and sex hormone-binding globulin (SHBG) in the Third National Health and Nutrition Examination Survey (NHANES III).

## Methods

### Study Population

NHANES III was a stratified, multistage probability survey designed to select a representative sample of the civilian non-institutionalized US population [17]. NHANES III included two phases (phase I: October 1988 – October 1991 and phase II: September 1991 – October 1994), each capable of independently producing unbiased national estimates. Within each phase, participants were randomly assigned to either a morning or afternoon/evening examination. The present study was conducted among men aged ≥ 20 years participating in the morning examination session of phase I (N = 1,967). The study was restricted to participants in the morning session to reduce extraneous variation due to diurnal production of sex hormones. Serum for hormone assays was available for 1,470 participants (75%). After excluding 25 participants missing data for urinary cadmium, 11 participants missing data for urinary creatinine, 7 participants missing data for serum testosterone, 5 participants missing data for serum SHBG, 9 participants missing data for serum albumin, 11 participants missing data for serum cotinine, and 140 participants missing data for other covariates, the final sample included 1,262 men. The protocol for NHANES III was approved by the National Center for Health Statistics of the Center for Disease Control and Prevention Institutional Review Board. All participants gave written informed consent. The assay of stored serum specimens for the Hormone Demonstration Program was approved by the Institutional Review Boards at the Johns Hopkins Bloomberg School of Public Health and the National Center for Health Statistics, Centers for Disease Control and Prevention.

### Data Collection

Demographic, household income, physical activity, cigarette smoking, and alcohol consumption data were collected using standardized questionnaires during an in-home interview [17]. Whole body electrical resistance was measured using a Valhalla Scientific Body Composition Analyzer (model 1990B; Valhalla Scientific, Inc., San Diego, CA) and prediction equations were used to predict percent body fat [18]. Serum cotinine was measured using liquid chromatography/atmospheric pressure ionization tandem mass spectrometry [19]. Atomic absorption spectrometry was used to measure blood lead as described by Sassa and colleagues [20] and serum selenium as described by Lewis and colleagues [21].

All materials used for collecting and processing urinary cadmium specimens were screened for possible cadmium contamination [22]. A spot urine specimen was collected and shipped at -20°C to the NHANES laboratory at the National Centers for Environmental Health at the Centers for Disease Control and Prevention in Atlanta, GA. Urinary cadmium was measured by graphite furnace atomic absorption with Zeeman background correction, using the CDC modification of the method of Pruszkowska and colleagues [23]. Since NHANES III only collected spot urine samples, all analyses were performed using creatinine corrected urinary cadmium values (urinary cadmium divided by urinary creatinine concentrations, expressed as μg/g) to account for between participant differences in urine dilution. Urinary creatinine was measured using the method of Jaffe with a Beckman ASTRA automated analyzer [24].

### Sex Steroid Hormones

Participants in the morning examination session fasted overnight before having blood drawn. After centrifugation, serum was aliquotted and stored at -70°C. Serum concentrations of testosterone, estradiol, AAG (a metabolite of dihydrotestosterone), and SHBG were measured in 2005 at the laboratory of Dr. Nader Rifai at Children's Hospital in Boston, MA. Sex steroid hormone levels and SHBG are stable after multiple freeze-thaw cycles [25,26]. Testosterone, estradiol, and SHBG levels were quantified using competitive electrochemiluminescence immunoassays on the 2010 Elecsys autoanalyzer (Roche Diagnostics, Indianapolis, IN). AAG was measured by an enzyme immunoassay (Diagnostics Systems Laboratories, Webster, TX). The detection limits of the assays were 0.02 ng/mL for testosterone, 5 pg/mL for estradiol, 0.33 ng/mL for AAG, and 3 nmol/L for SHBG. The coefficients of variation (CV%) for quality control specimens included during the analyses of the NHANES III samples ranged from 5.8 to 5.9% for testosterone, 2.5 to 6.7% for estradiol, 5.0 to 9.5% for AAG, and 5.3 to 5.9% for SHBG. Free testosterone concentrations were estimated from measured testosterone, SHBG, and albumin, while free estradiol concentrations were estimated from measured estradiol, SHBG, and albumin [27,28].

### Statistical Analysis

Participants were categorized into quartiles of urinary cadmium based on the weighted population distribution. Age and race-ethnicity adjusted means and percentages were calculated by linear regression for continuous covariates and logistic regression for dichotomous covariates.

Due to skewed distributions of sex steroid hormones, we calculated adjusted geometric means by quartile of cadmium using multiple linear regression on log-transformed hormone levels. We also present analyses for molar ratios of estradiol to testosterone, testosterone to SHBG, and estradiol to SHBG. Initial models adjusted for age and race-ethnicity. Subsequent models further adjusted for smoking status and serum cotinine. Additional models further adjusted for household income, physical activity, alcohol consumption, percent body fat, blood lead, and serum selenium. Tests for linear trend across quartiles of cadmium were computed by including an ordinal variable with the median of each quartile of cadmium in the linear regression models. Due to the importance of smoking in determining cadmium levels and the possibility of overadjustment, we additionally present models separately for never, former, and current smokers.

Data were analyzed using SUDAAN (version 9.0; Research Triangle Institute, Research Triangle Park, NC) to account for the complex NHANES sampling design, including unequal probabilities of selection, over-sampling, and non-response.

## Results

The median level (range) of urinary cadmium in the study sample was 0.34 μg/g creatinine (0.003–4.22 μg/g). Participants with higher cadmium levels were more likely to be older, to be non-Hispanic black, to be current smokers, to have household incomes < $20,000, not to exercise, and to have higher blood lead levels (Table 1). The median levels (range) of urinary cadmium among never (n = 436), former (n = 416), and current (n = 410) smokers were 0.21 μg/g (0.003–4.22 μg/g), 0.42 μg/g (0.004–2.32 μg/g), and 0.56 μg/g (0.004–4.02 μg/g), respectively.

**Table 1:** Age and race-ethnicity adjusted participant characteristics* by quartile of creatinine corrected urinary cadmium

	Quartile 1 < 0.18 μg/g	Quartile 2 0.18–0.33 μg/g	Quartile 3 0.34–0.62 μg/g	Quartile 4 ≥ 0.63 μg/g	p-trend
Age, years	31.7 (0.8)	37.1 (0.7)	44.6 (1.3)	54.3 (1.2)	< 0.001
Non-Hispanic white, %	83.9 (2.9)	80.3 (3.4)	74.5 (5.6)	74.3 (5.2)	0.07
Non-Hispanic black, %	6.5 (1.5)	10.9 (2.2)	8.1 (1.1)	11.1 (1.7)	0.03
Mexican-American, %	4.2 (1.0)	5.6 (1.0)	4.5 (0.9)	4.7 (0.8)	0.99
Current smokers, %	11.2 (2.1)	20.4 (3.7)	42.9 (4.2)	73.6 (2.4)	< 0.001
Former smokers, %	29.3 (4.3)	33.9 (4.2)	35.8 (3.7)	25.7 (3.2)	0.06
Serum cotinine, ng/mL	26.4 (8.4)	39.6 (8.4)	107.2 (12.9)	231.0 (12.1)	< 0.001
Low income, %	24.1 (3.0)	20.4 (2.7)	31.0 (2.9)	42.1 (4.0)	< 0.001
Consume alcohol, %	75.6 (4.1)	71.5 (3.4)	74.8 (3.2)	72.7 (3.8)	0.76
No exercise, %	40.6 (6.1)	30.1 (3.5)	41.0 (4.4)	55.2 (4.2)	0.005
Percent body fat, mean percent	25.8 (0.5)	26.1 (0.5)	25.4 (0.6)	24.7 (0.5)	0.05
Blood lead, μg/dL†	3.2 (2.8, 3.6)	3.6 (3.2, 4.0)	4.3 (3.9, 4.8)	5.5 (4.9, 6.3)	< 0.001
Serum selenium, ng/mL	126.5 (2.7)	126.7 (1.1)	127.3 (1.3)	121.7 (1.3)	0.05

*Mean or percentage (standard error)

†Geometric mean (95% confidence interval)

After adjustment for age and race-ethnicity, higher cadmium levels were associated with higher levels of total testosterone, total estradiol, SHBG, estimated free testosterone, and estimated free estradiol (each p-trend < 0.05), but not with AAG or any of the molar ratios (Table 2). After additionally adjusting for smoking status and serum cotinine, the associations of urinary cadmium levels with sex steroid hormones and their molar ratios were small and not statistically significant (each p-trend > 0.05). After stratifying by smoking status, the associations of urinary cadmium levels with sex steroid hormones and their molar ratios were small and not statistically significant for never, former, and current smokers (each p-trend > 0.05; Table 3). However, among never smokers there was a marginally significant, negative association between urinary cadmium and total testosterone (p-trend = 0.06) and among current smokers there was a marginally significant, positive association between urinary cadmium and free testosterone concentrations (p-trend = 0.09).

**Table 2:** Adjusted geometric means (95% confidence interval) by quartile of creatinine corrected urinary cadmium

	Quartile 1 < 0.18 μg/g	Quartile 2 0.18–0.33 μg/g	Quartile 3 0.34–0.62 μg/g	Quartile 4 ≥ 0.63 μg/g	p-trend
Total testosterone, ng/mL					
Age and race-ethnicity adjusted	4.69 (4.40, 4.99)	4.85 (4.45, 5.27)	5.13 (4.78, 5.52)	5.64 (5.27, 6.04)	< 0.001
Multivariable model 1†	5.06 (4.78, 5.36)	5.06 (4.71, 5.44)	5.03 (4.68, 5.41)	5.09 (4.69, 5.53)	0.88
Multivariable model 2‡	5.10 (4.85, 5.35)	5.07 (4.72, 5.45)	5.00 (4.67, 5.35)	5.08 (4.68, 5.51)	0.99
Total estradiol, pg/mL					
Age and race-ethnicity adjusted	32.1 (29.6, 34.8)	34.6 (33.1, 36.1)	37.5 (35.8, 39.2)	39.8 (37.9, 41.8)	< 0.001
Multivariable model 1†	34.4 (32.2, 36.8)	36.1 (34.6, 37.7)	36.9 (35.2, 38.6)	36.2 (33.8, 38.7)	0.42
Multivariable model 2‡	34.3 (32.3, 36.4)	36.0 (34.5, 37.6)	37.1 (35.4, 38.8)	36.1 (33.7, 38.8)	0.41
SHBG, nmol/L					
Age and race-ethnicity adjusted	34.2 (31.4, 37.2)	33.1 (30.9, 35.4)	34.6 (32.6, 36.7)	37.5 (34.6, 40.7)	0.009
Multivariable model 1†	35.2 (32.1, 38.6)	33.9 (31.6, 36.3)	34.4 (32.3, 36.7)	35.6 (32.7, 38.8)	0.57
Multivariable model 2‡	35.5 (32.7, 38.5)	34.0 (31.7, 36.4)	34.3 (32.4, 36.3)	35.4 (32.5, 38.5)	0.71
Androstanediol glucuronide, ng/mL					
Age and race-ethnicity adjusted	13.0 (12.2, 13.8)	11.8 (10.9, 12.9)	10.9 (9.5, 12.5)	11.2 (9.7, 13.0)	0.23
Multivariable model 1†	12.9 (12.1, 13.8)	11.8 (10.8, 12.9)	10.9 (9.5, 12.5)	11.3 (9.8, 13.1)	0.33
Multivariable model 2‡	12.9 (12.1, 13.7)	11.7 (10.8, 12.8)	10.9 (9.5, 12.5)	11.4 (9.8, 13.2)	0.40
Estimated free testosterone, ng/mL					
Age and race-ethnicity adjusted	0.092 (0.087, 0.097)	0.098 (0.091, 0.105)	0.104 (0.095, 0.113)	0.109 (0.102, 0.118)	< 0.001
Multivariable model 1†	0.099 (0.094, 0.105)	0.102 (0.095, 0.108)	0.101 (0.093, 0.111)	0.100 (0.091, 0.109)	0.89
Multivariable model 2‡	0.100 (0.094, 0.105)	0.102 (0.095, 0.109)	0.101 (0.092, 0.110)	0.100 (0.090, 0.110)	0.85
Estimated free estradiol, pg/mL					
Age and race-ethnicity adjusted	0.81 (0.75, 0.89)	0.89 (0.84, 0.93)	0.96 (0.91, 1.02)	1.00 (0.94, 1.07)	< 0.001
Multivariable model 1†	0.87 (0.81, 0.94)	0.92 (0.87, 0.97)	0.95 (0.90, 1.00)	0.91 (0.84, 0.99)	0.69
Multivariable model 2‡	0.87 (0.81, 0.93)	0.92 (0.87, 0.97)	0.95 (0.90, 1.01)	0.91 (0.83, 1.00)	0.69
Estradiol*1000/total testosterone					
Age and race-ethnicity adjusted	7.24 (6.57, 7.99)	7.56 (6.81, 8.39)	7.74 (7.18, 8.34)	7.47 (6.98, 8.00)	0.80
Multivariable model 1†	7.20 (6.55, 7.91)	7.55 (6.83, 8.34)	7.75 (7.14, 8.42)	7.52 (6.90, 8.20)	0.70
Multivariable model 2‡	7.12 (6.60, 7.69)	7.52 (6.80, 8.31)	7.85 (7.23, 8.53)	7.54 (6.86, 8.28)	0.61
Total testosterone/SHBG					
Age and race-ethnicity adjusted	0.476 (0.445, 0.510)	0.508 (0.475, 0.543)	0.514 (0.469, 0.564)	0.522 (0.480, 0.566)	0.05
Multivariable model 1†	0.498 (0.461, 0.538)	0.518 (0.488, 0.551)	0.507 (0.460, 0.558)	0.495 (0.444, 0.553)	0.71
Multivariable model 2‡	0.498 (0.461, 0.538)	0.518 (0.485, 0.554)	0.505 (0.458, 0.555)	0.497 (0.442, 0.559)	0.79
Estradiol*1000/SHBG					
Age and race-ethnicity adjusted	3.45 (3.03, 3.92)	3.84 (3.52, 4.20)	3.98 (3.66, 4.32)	3.90 (3.49, 4.35)	0.15
Multivariable model 1†	3.59 (3.14, 4.09)	3.91 (3.56, 4.30)	3.93 (3.60, 4.28)	3.72 (3.29, 4.22)	0.98
Multivariable model 2‡	3.55 (3.16, 3.99)	3.90 (3.55, 4.28)	3.97 (3.66, 4.30)	3.75 (3.29, 4.27)	0.87

SHBG = Sex hormone-binding globulin

†Adjusted for age (continuous) and race-ethnicity (non-Hispanic white, non-Hispanic black, Mexican-American, and other), smoking status (never, former, and current), and serum cotinine (continuous)

‡Adjusted for variables in model 1 and household income (< $20,000 and ≥ $20,000), physical activity (none, 1–2, and ≥ 3 times a week), alcohol consumption (< 12 and ≥ 12 drinks in the past year), percent body fat (continuous), blood lead (log-transformed, continuous), and serum selenium (continuous)

**Table 3:** Adjusted* geometric means (95% confidence interval) by quartile of creatinine corrected urinary cadmium and smoking status.

	Quartile 1 < 0.18 μg/g	Quartile 2 0.18–0.33 μg/g	Quartile 3 0.34–0.62 μg/g	Quartile 4 ≥ 0.63 μg/g	p-trend
Total testosterone, ng/mL					
Never smokers	5.03 (4.68, 5.41)	4.88 (4.27, 5.58)	4.12 (3.26, 5.22)	4.24 (3.24, 5.55)	0.06
Former smokers	4.34 (3.91, 4.81)	4.16 (3.60, 4.80)	4.52 (4.20, 4.87)	4.49 (4.05, 4.98)	0.49
Current smokers	5.74 (5.08, 6.48)	5.78 (5.34, 6.27)	6.09 (5.81, 6.39)	6.39 (5.86, 6.96)	0.12
Total estradiol, pg/mL					
Never smokers	31.7 (29.0, 34.6)	34.0 (31.4, 36.7)	34.3 (30.8, 38.2)	30.2 (23.6, 38.7)	0.97
Former smokers	33.0 (30.1, 36.2)	33.0 (31.0, 35.0)	32.6 (30.8, 34.4)	33.5 (31.0, 36.2)	0.73
Current smokers	40.2 (36.3, 44.5)	39.1 (37.0, 41.3)	44.3 (42.3, 46.5)	43.2 (40.7, 45.8)	0.26
SHBG, nmol/L					
Never smokers	32.4 (29.6, 35.3)	32.6 (29.8, 35.6)	31.3 (27.1, 36.1)	32.5 (26.8, 39.3)	0.88
Former smokers	36.2 (30.6, 42.8)	32.2 (29.7, 34.8)	37.3 (33.5, 41.7)	37.8 (34.1, 41.8)	0.28
Current smokers	39.3 (34.7, 44.5)	36.3 (31.7, 41.6)	33.7 (31.0, 36.6)	37.6 (33.2, 42.7)	0.71
Androstanediol glucuronide, ng/mL					
Never smokers	12.9 (12.0, 13.8)	11.3 (9.9, 13.0)	11.7 (9.7, 14.0)	13.1 (11.0, 15.7)	0.80
Former smokers	14.0 (11.8, 16.7)	11.8 (10.3, 13.6)	10.5 (9.7, 11.3)	11.7 (9.6, 14.4)	0.64
Current smokers	12.1 (9.8, 14.9)	12.5 (10.0, 15.4)	10.8 (8.7, 13.3)	11.0 (9.3, 12.9)	0.53
Estimated free testosterone, ng/mL					
Never smokers	0.102 (0.094, 0.111)	0.099 (0.088, 0.111)	0.084 (0.063, 0.112)	0.085 (0.064, 0.114)	0.14
Former smokers	0.083 (0.075, 0.091)	0.084 (0.071, 0.099)	0.086 (0.079, 0.093)	0.084 (0.076, 0.093)	0.97
Current smokers	0.109 (0.097, 0.123)	0.114 (0.106, 0.123)	0.129 (0.121, 0.137)	0.126 (0.117, 0.136)	0.09
Estimated free estradiol, pg/mL					
Never smokers	0.81 (0.73, 0.90)	0.86 (0.79, 0.94)	0.89 (0.78, 1.00)	0.77 (0.60, 1.00)	0.92
Former smokers	0.84 (0.75, 0.94)	0.86 (0.80, 0.92)	0.82 (0.78, 0.87)	0.84 (0.77, 0.91)	0.87
Current smokers	1.00 (0.90, 1.10)	0.99 (0.92, 1.05)	1.16 (1.09, 1.23)	1.09 (1.01, 1.17)	0.39
Estradiol*1000/total testosterone					
Never smokers	6.67 (6.06, 7.35)	7.37 (6.08, 8.93)	8.81 (6.75, 11.50)	7.54 (6.20, 9.17)	0.16
Former smokers	8.08 (6.96, 9.37)	8.41 (7.36, 9.60)	7.62 (7.02, 8.28)	7.89 (7.10, 8.77)	0.71
Current smokers	7.41 (6.75, 8.14)	7.15 (6.62, 7.72)	7.71 (7.25, 8.19)	7.16 (6.58, 7.79)	0.51
Total testosterone/SHBG					
Never smokers	0.539 (0.490, 0.593)	0.520 (0.470, 0.575)	0.457 (0.337, 0.619)	0.453 (0.332, 0.619)	0.25
Former smokers	0.416 (0.364, 0.474)	0.448 (0.382, 0.525)	0.420 (0.380, 0.464)	0.412 (0.370, 0.459)	0.59
Current smokers	0.507 (0.456, 0.563)	0.552 (0.491, 0.621)	0.628 (0.575, 0.685)	0.588 (0.535, 0.646)	0.31
Estradiol*1000/SHBG					
Never smokers	3.60 (3.09, 4.19)	3.83 (3.37, 4.36)	4.03 (3.29, 4.93)	3.42 (2.62, 4.45)	0.88
Former smokers	3.35 (2.70, 4.15)	3.77 (3.37, 4.20)	3.20 (2.90, 3.53)	3.25 (2.86, 3.70)	0.52
Current smokers	3.75 (3.30, 4.27)	3.95 (3.44, 4.53)	4.84 (4.35, 5.38)	4.22 (3.66, 4.86)	0.72

*Adjusted for age (continuous) and race-ethnicity (non-Hispanic white, non-Hispanic black, Mexican-American, and other), serum cotinine (continuous), household income (< $20,000 and ≥ $20,000), physical activity (none, 1–2, and ≥ 3 times a week), alcohol consumption (< 12 and ≥ 12 drinks in the past year), percent body fat (continuous), blood lead (log-transformed, continuous), and serum selenium (continuous)

## Discussion

In this large, representative sample of US adult men, urinary cadmium levels were not associated with sex steroid hormone levels after adjustment for confounders. In our analysis, smoking was an important confounder of the association between cadmium and sex steroid hormone levels. The lack of association between cadmium and sex steroid hormones was also evident after stratifying by smoking status, although we identified a marginally significant association between cadmium and total testosterone among never smokers and free testosterone among current smokers. These findings may be explained as chance findings or a result of residual confounding, although we cannot exclude a modest association.

Four previous studies have evaluated the association of urine or blood cadmium with sex steroid hormone levels in men [10-13]. In a cross-sectional study of Chinese men with environmental exposure to cadmium, blood and urine cadmium did not meet the criteria for inclusion in stepwise models predicting testosterone levels [10]. Blood cadmium was positively associated with testosterone in two separate studies of Croatian men who had never been occupationally exposed to cadmium [11,12] and in a study of Chinese male smelter workers [13]. These studies were relatively small: the largest study included 263 participants, and their combined sample size was 701. Additionally, three of these studies did not report smoking adjusted results [10-12], although smoking was included in stepwise regression algorithms. The importance of tobacco use as a confounder of the cadmium-sex hormone relationship may differ across populations due to varying levels of cadmium in tobacco, prevalence of tobacco use, and exposure to cadmium from other sources (e.g., occupational exposure, food, air pollution). However, our analysis indicates that studies of the association of cadmium with sex steroid hormones need to carefully control for tobacco use.

Our findings pertain to low-level chronic environmental cadmium exposure and may not be generalizable to environmental or occupational settings involving higher dosages of cadmium. Indeed, differences in cadmium levels may account for the inconsistent results in previous research. Additionally, urinary cadmium reflects predominantly cumulative long-term exposure and we cannot rule out short-term effects of cadmium exposure on sex steroid hormone levels. Our analysis was based on single measures of sex steroid hormones in plasma and of cadmium in spot urine samples, all of which were subject to substantial within person variability and laboratory measurement error. Consequently, we cannot exclude the existence of a modest association between urine cadmium and sex steroid hormone levels. However, the inconsistent findings in experimental models and the lack of a clearly delineated biological mechanism that could explain an effect of chronic environmental cadmium exposure on sex steroid hormone concentrations support our findings of no association.

## Conclusion

Cadmium binds with high affinity to the androgen receptor, where it inhibits the binding of androgens and induces androgen-like effects [29]. While cadmium itself may exert androgen-like effects, our analysis indicates that urinary cadmium is not an important determinant of sex steroid hormone levels in adult men at the range of exposure evaluated in the general US population.

## Abbreviations

AAG – androstanediol glucuronide; SHBG – sex hormone-binding globulin; NHANES III – Third National Health and Nutrition Examination Survey

## Competing interests

The author(s) declare that they have no competing interests.

## Authors' contributions

AM, EG, MSS, and EAP were responsible for the conception and design, analysis and interpretation of data. AM drafted the manuscript, that was edited by EG, MSS, and EAP. SR, SB, NR, and WGN were responsible for interpretation of the data and revising the manuscript. All authors read and approved the final manuscript.

## Pre-publication history

The pre-publication history for this paper can be accessed here:


